# Supplementary material for: Fast light-field 3D microscopy with out-of-distribution detection and adaptation through Conditional Normalizing Flows
Source: ArXiv. 2023 Jun 14:arXiv:2306.06408v2. Preprint. [Version 2] (PMC10312789)
Supplement: 1 [file NIHPP2306.06408V2-supplement-1.pdf]

arXiv:2306.06408v2 [eess.IV] 14 Jun 2023

Supplementary document: Fast light-field 3D microscopy, out of distribution detection and adaptation through Conditional Normalizing Flows

1. THE CONDITIONAL WAVELET FLOW ARCHITECTURE

We augmented the Freia framework [**?**] for better handling of our problem in hand. Some of the modifications are:

- Permutation along random dimensions: The permutation operation is typically done only on the channel dimension. We modified the implementation to randomly select a dimension to permute and apply a random permutation of the elements on that dimension.
- 1D Haar transform: The original Wavelet-Flow paper [**?**] up/down sampled the images on the x-y axes, however in the case of XLFM images, the lenslet images already contain the high definition information in x-y dimension. Hence, compressing the images to upsample them again seemed counter intuitive. Instead, we perform the up/down sampling along the channel dimension. We are starting with 8 depths and upsampling with 4 CWFs until reaching 96 depths.

Additionally, instead of the traditional permutation functions, where the channel dimension is permuted, we included permutations where other dimensions can be permuted. See FIg. 1 for details on the internal components of the network.

2. LOW RESOLUTION 3D RECONSTRUCTION (LR-NET)

LL-net shown in Fig. S4a, performs a low axial resolution reconstruction with an XLFM image and low-resolution mean volume as input. It is comprised of the following parts:

- The perspective views corresponding to each microlens is cropped and stacked on the channel dimension.
- The views are passed to a 2D unit, where the channel dimension encodes the volume's axial dimension.
- In parallel, the mean volume is passed through two ConvNext convolutional blocks and a global attention module in a residual multiplicative matter.
- The global attention module selects the relevant pixel-wise information by first turning the image into a 1D array, then applying a Conv1D, a ReLU, a Conv1d, and a sigmoid activation which outputs a value from 0 to 1 that gets multiplied or weights the original pixel.
- the U-net and ConvNext paths are added into a single volume. Resulting in a $512 \times 512 \times \frac{96}{2^n}$ voxels, where n is the number of down-sampling steps in the CWFA (5 in our case).

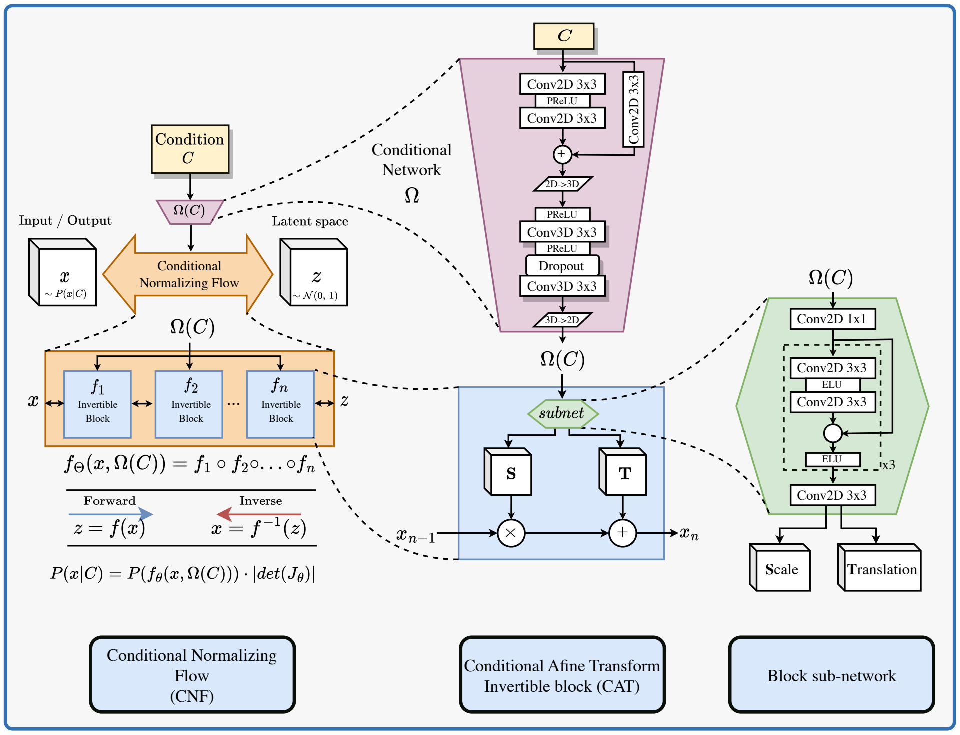

Fig. S1. Single conditional normalizing flow used within the CFWA, also present in Fig. **??** as CNF1,2, etc. In blue, the CAT block is responsible for computing a scaling and translation from the condition and applying it to the input

Description	Age	Cross-val fold test
NLS GCaMP6s	unknown age	0
NLS GCaMP6s	unknown age	1
Pan-neuronal nuclear localized GCaMP6s Tg(HuC:H2B:GCaMP6s)	4-6 days	2
Pan-neuronal nuclear localized GCaMP6s Tg(HuC:H2B:GCaMP6s)	4-6 days	3
Soma localized GCaMP7f Tg(HuC:somaGCaMP7f)	4-6 days	4
Soma localized GCaMP7f Tg(HuC:somaGCaMP7f)	4-6 days	5

Table S1. Description of fish used for this work and in which cross-validation set they were used as testing set.

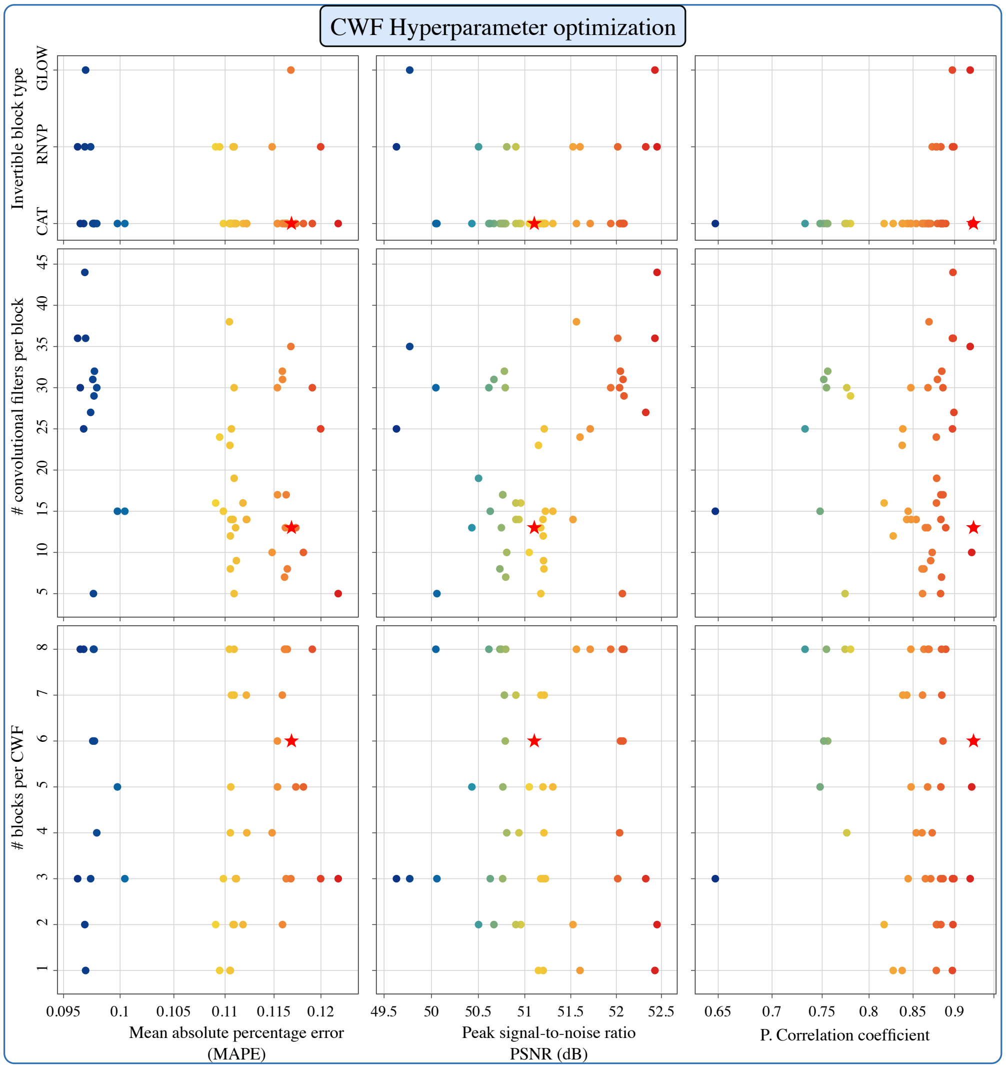

Fig. S2. Hyper-parameter ablation optimized towards Pearson correlation coefficient. Highlighted with a red star is the selected architecture.

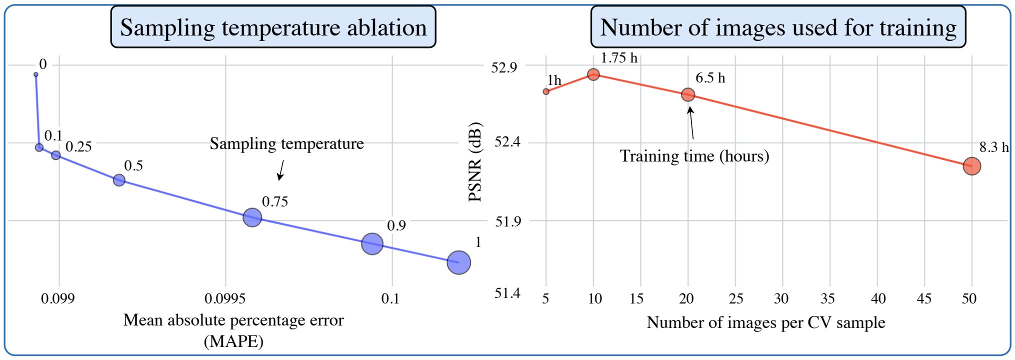

Fig. S3. (Left) Sampling temperature ablation. (Right) The number of samples used per CV set during training. Both were evaluated on CV fold 0. We performed a similar evaluation fo the WF parameters and the LR-Net.

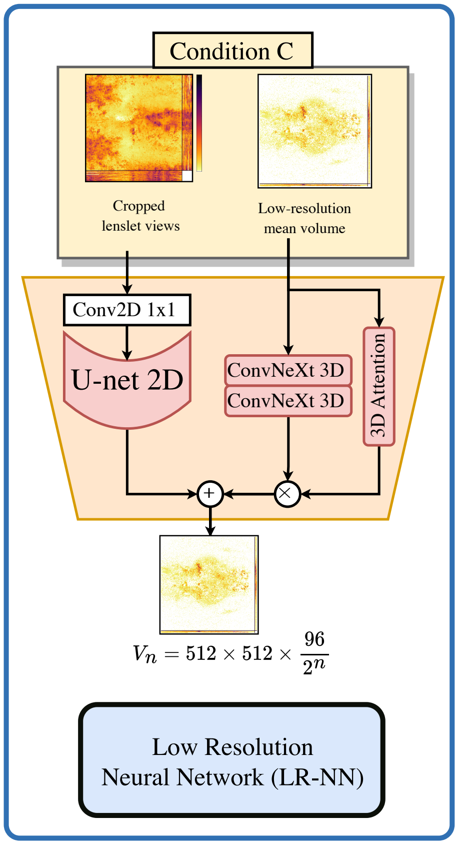

(a) Low-resolution reconstruction network, clarified in sec. 2, used as an initial step for the reconstruction operation. Where the stacked lenslet views of the 2D XLFM image and a mean low-resolution volume are used as input. And a 3D volume with low axial resolution results as an output. Later upsampled by the remaining CWF blocks in the CWFA network.

CWFA step	NLL threshold	F1-score	AUC
1	-1.33	0.9933	0.9985
2	-0.65	0.9886	0.9933
3	-0.21	0.9402	0.9647
4	-0.26	0.9615	0.9833

(b) Out-of-distribution detection scores for all CWF steps.

Metric	Before fine-tune	Fine-tune only testing DS	% ⇑	Fine-tune all DSs	% ⇑
PSNR	39.1584	53.4765	36.5644%	52.6036	34.3352%
MAPE	0.1492	0.0884	40.7160%	0.0984	34.0131%
PCC	0.8944	0.9137	2.1578%	0.9065	1.3528
Time	-	5 min.	-	25 min.	-

Table S2. Improvement upon fine-tuning on 10 images of the testing set (column 3-4). Or by appending the 10 images to the cross-validation training set (column 5-6).

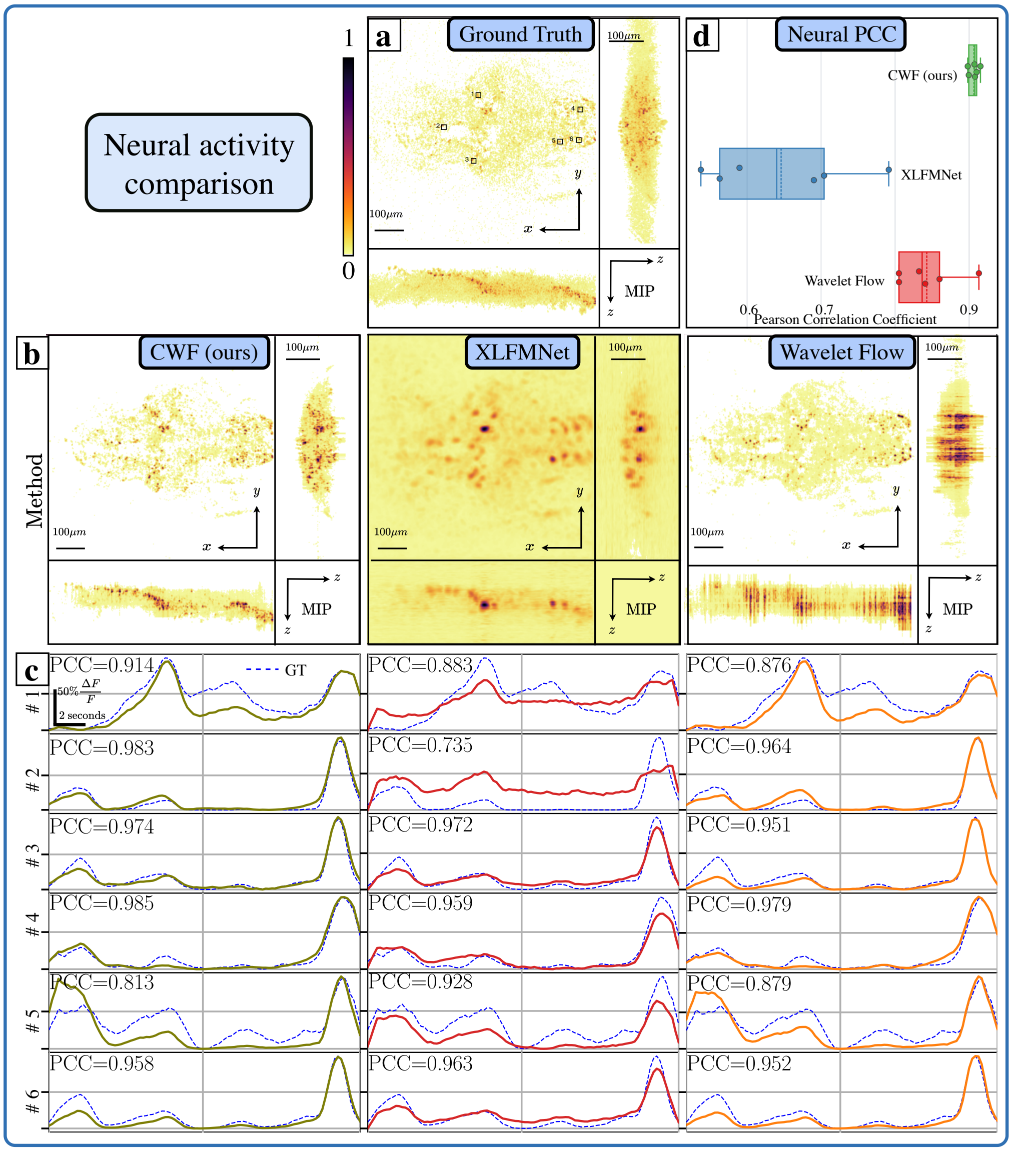

Fig. S5. Neural activity comparison with different methods. In **(a)**, the MIP of the GT volume, with a subset of the active neurons highlighted. Followed by a reconstructed frame with different methods in row **(b)**. In **(c)**, the neural potentials of 6 neurons in 100 frames (10 seconds). (d) the mean Pearson correlation coefficient with six fish and the three methods are shown. Note that the PCC was measured directly on the 3D volumes, and only the 2D projection of the neuron coordinates are shown in **(a)**
